# Second trimester hepatic rupture in a 35 year old nulliparous woman with HELLP syndrome: a case report

**DOI:** 10.1186/1749-7922-4-23

**Published:** 2009-06-15

**Authors:** J Kelly, DJ Ryan, N O'Brien, WO Kirwan

**Affiliations:** 1 Department of Surgery, Cork University Hospital, Wilton, Cork, Ireland

## Abstract

The HELLP syndrome (haemolysis, elevated liver blood tests and low platelets) is a serious complication in pregnancy characterized by haemolysis, elevated liver enzymes and low platelet count occurring in 0.5 to 0.9% of all pregnancies and in 10–20% of cases with severe preeclampsia. Hepatic capsular rupture is a rare yet dramatic complication of HELLP syndrome. The majority of cases occur in multiparous women over the age of 30. Classically it presents with acute onset right upper quadrant pain in the presence of constitutional symptoms such as vomiting and pyrexia. However, symptoms and signs are usually non specific. Spontaneous hepatic rupture can be preceded by signs of hypovolaemic shock; yet the diagnosis is infrequently made prior to emergent laparotomy. We present the case of a 35 year old nulliparous woman with a second trimester gestational hepatic rupture associated with HELLP syndrome. We briefly discuss the aetiology, diagnostic difficulties and treatment options associated with this rare presentation.

## Case presentation

A previously healthy 35 year old nulliparous woman conceived secondary to egg donation in-vitro fertilisation therapy on a background of primary infertility. Routine antenatal booking visit at 14 weeks gestation revealed a blood pressure of 146/81 with a normal urine specimen. At 18 weeks gestation, she was found to have +3 proteinuric asymptomatic hypertension (184/102 mm Hg) with HELLP syndrome [platelets 105 (150–400 × 10^9 ^per litre), alanine transaminase 2223 (5–40 IU/L), aspartate transaminase 2823 (10–40 IU/L), lactate dehyrogenase 14361(> 600 U/L), INR 1.6 (<1.0), activated partial thromboplastin time 186 (25–40 secs) and a 24 hour urine collection showed 2.8 gr of protein. She complained of some mild epigastric discomfort, but this settled with simple analgesia. She was promptly commenced on anti-hypertensive medicine.

Her anti – hypertensive requirements gradually increased with an observable worsenening of peripheral oedema and proteinuria. Radiological investigations inclusive of ultrasound of kidneys, gallbladder, spleen and liver at that time were all normal. Multi-disciplinary investigation of underlying aetiologies for this early onset pre-eclampsia did not discern a cause. Connective tissue screening was negative.

**Figure 1:**
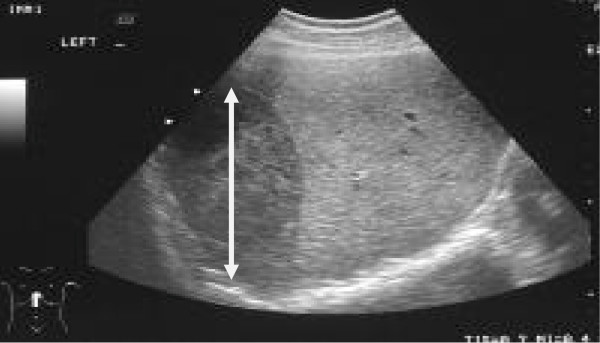
**Liver ultrasound shows large haematoma (white arrow spanning the length of the hyperechoic area representing fresh blood)**.

Although a normal multi-vessel Doppler was present, the estimated fetal weight was 184 grams (<3rd percentile). Two days post admission the patient's condition changed. She became acutely haemodynamically unstable complaining of severe epigastric pain and obvious hyperreflexia. Immediate transfer to the High Dependency Unit occurred. Ultrasound scan revealed a large liver haematoma (figure 1). The fetal heart beat was still present. She received 4 units of O negative blood. A repeat ultrasound one hour later revealed free blood in the abdominal cavity; the fetal heart beat was now absent.

**Figure 2:**
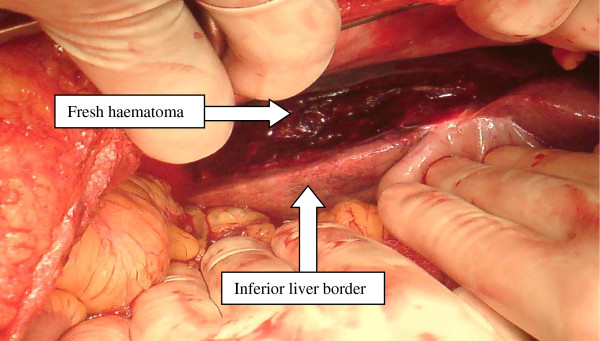
**Intraoperative finding of a large liver haematoma overlying the infero-lateral border of the liver**.

The patient was immediately transferred to the operating theatre for a laparotomy. The abdomen was opened through a midline incision. The bleeding was found to be emanating from a ragged laceration on the anterior aspect of the right lobe of the liver which was fully accessible without the need for mobilisation of the liver (figure 2). Coagulopathy prevented haemostasis by electro-cautery or using topical agents and thus haemostasis was secured by packing the liver with gauze swabs placed above and around the liver in a routine manner. A hysterotomy and removal of a non-viable fetus was also performed. The abdomen was closed with interrupted PDS sutures to the fascia and clips to the skin without undue difficulty. A second-look laparotomy was performed at 48 hours at which stage the swabs were removed and a liver biopsy taken with a Tru-cut biopsy needle. There was no evidence of abdominal compartment syndrome at any stage.

Her post operative course was eventful in that she developed multi-organ failure requiring a two week stay in the intensive care unit with renal replacement therapy, mechanical ventilation and vasopressor support. Fortunately, she made a prompt recovery and was discharged home on day 20. She was counselled against attempting to get pregnant again in view of the risk of recurrence of the HELLP syndrome.

**Figure 3:**
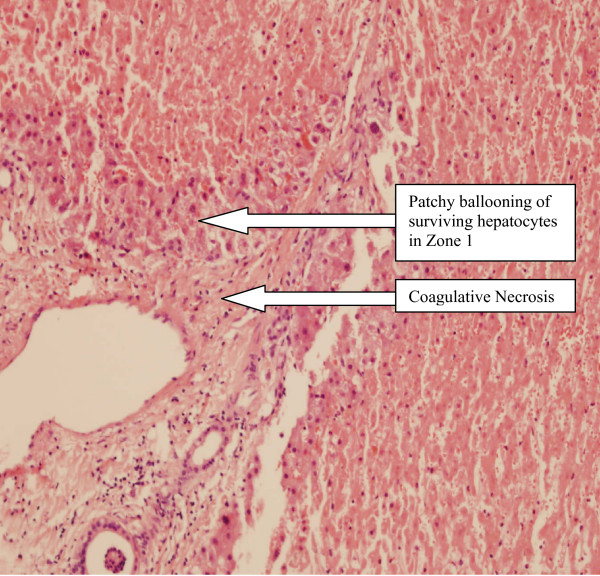
**Hepatic biopsy showing patchy ballooning of surviving hepatocytes in Zone 1 and coagulative necrosis**.

Hepatic biopsy revealed massive hepatic necrosis explaining the patients liver failure (figure 3).

## Discussion

With only 200 cases of hepatic rupture documented in the global literature, it is not surprising that few doctors have experience in dealing with this condition [1].

### Aetiology

In the Tennessee Classification System, diagnostic criteria for HELLP are haemolysis with increased LDH (> 600 U/L), AST (≥ 70 U/L), and platelets < 100 × 10^9^/L. The pathophysiology of this condition is complex and poorly understood. The origin of pre-eclampsia/HELLP can be attributed to defective trophoblastic invasion. As a consequence of this trophoblastic dysfunction, a desirable high flow, low resistance circuit for adequate placental function fails to develop. It appears that the fundamental component of this situation is abnormal placental cyclo-oxygense activity. COX 1 activity remains the same in the placenta however, COX 2 expression is decreased [2]. The net result of this is preferential production of thromboxane, a potent vasoconstrictor and mediator of platelet aggregation over prostacyclin. As a consequence of this vasoconstrictive stimulus, and increased afterload on the heart secondary to uteroplacental dysfunction, mean arterial pressure increases.

Hypertension, in addition to thromboxane causes endothelial dysfunction in the maternal vasculature particularly in the organs with highest blood flow (liver, kidneys, brain). Fibrin plus platelet deposits further stenose vessels that are currently vasospastic. This vasospasm is excellently highlighted on examination of the cerebral vasculature where blood flow velocity is increased in patients with pre-eclampsia/HELLP syndrome as illustrated by transcranial Doppler studies [3]. Autoregulation of blood pressure occurs between 60–150 mmHg. In response to raised BP, vasospasm occurs in an attempt to decrease MAP. Clinical effects of this vasospasm have been illustrated in case reports causing a diversity of effects such as hemiparesis [4], optical ataxia and transient cortical blindness [5] depending on the cerebral vessel affected. The importance of these vasospastic segments is that they serve as a nidus for microangiopathic haemolytic anaemia [6].

Oxy Hb is a potent vasoconstrictor [7] perpetuating the cycle and causes effects such as hepatic infarction. The vasoconstrictive effect of oxy Hb can be attributed to its ability to inhibit the production of endothelial derived relaxing factor (EDRF). In the kidneys this vasospasm, with superimposed microthromi reduce glomerular filtration rate and result in acute tubular necrosis [8].

Vasospasm clearly results in hypoxia in the distal tissues. The effect of this is hypoxic induced angiogenesis. However these vessels are structurally much weaker than there existing counterparts. With haemolysis of the red blood cells, blood viscosity reduces and according to Poseuilles law there is increased flow and increased pressure. These new vessels formed in response to the hypoxic stimulus cannot contain the elevated flow and pressure, so rupture causing effects such as liver capsular haematomas which can result in hepatic capsular rupture [9].

### Diagnosis

In the Tennessee Classification System diagnostic criteria for HELLP are haemolysis with increased LDH (> 600 U/L), AST (≥ 70 U/L), and platelets < 100 × 10^9^/L.

The diagnosis of hepatic haematomas secondary to rupture is outlined in the *Annals of Hepatology *[1]. The most interesting point from their recommendations is that of a multidisciplinary approach in all stages of management. Radiologically liver ultrasound is the best screening tool. This should be performed if patients with HELLP complain of epigastric, right upper quadrant or shoulder tip pain in the presence of hypotension [10]. Antenatally Magnetic Resonance Imaging can further delineate the pathology, CT being preferable post natally. If angiography is available this modality can show the active point of bleeding being diagnostic and therapeutic. However if ultrasound reveals a hepatic haematoma with free fluid in the abdomen then immediate resuscitation with transfer for emergent laparotomy should occur. One third of patients with hepatic rupture die in haemorrhagic shock [11].

### Treatment

Although an Obstetric condition by its nature, the surgeons are the most frequently involved in the treatment of this condition. At laparotomy packing with abdominal towels is the usual means of haemostatic control. Also documented in the literature is the use of polypropylene mesh. If deemed appropriate the hepatic tear may be sutured and in some cases to achieve local haemostasis ligation of the hepatic artery is necessary. Surgical repair of the liver is quite different in the setting of fulminant HELLP syndrome due to the addition of impaired clotting and low platelets. Following tamponade, abdominal closure is recommended [4].

The haematologist's advice should be sought regarding blood transfusion, use of blood concentrates and platelets. A second look operation is performed after circa two days once haemodynamic and metabolic stabilisation has occurred. If haemostasis has not occurred repacking is the usual surgical option with/without the administration of fibrinolysis inhibitors such as aprotinin and anti-thrombin III.

Other less frequently used treatment modalities include activated factor VII [12], selective transarterial embolisation, partial liver resection, argon laser coagulation [13] and liver transplantation.

### Liver Transplantation

This is the most recent and promising development in the management of complicated HELLP syndrome. Orthotopic liver transplantation should be considered in the setting of uncontrollable haemorrhage, acute liver failure or macroscopic liver necrosis [14]. Of thirteen documented cases in the literature, ten made a successful recovery [6,15]. The three deaths occurred within 7 weeks of transplantation from prolonged sepsis. With such favourable statistics, it should be a viable option when treating such high risk patients.

## Conclusion

Although gestational hepatic rupture is a rare complication of preeclampsia, a high index of suspicion should exist when treating these patients with a focus at all times on multidisciplinary care. Although classically a condition with a mortality reaching as high as 85%, some centres boast a combined maternal – fetal mortality of 25%, reflecting the aforementioned changes in the diagnosis and treatment of this condition [16]. We contribute our favourable outcome to a multidisciplinary approach in all stages of management.

## Consent

Written informed consent was obtained from the patient for publication of this case report and any accompanying images. A copy of the written consent is available for review by the Editor-in-Chief of this journal.

## Competing interests

The authors declare that they have no competing interests.

## Authors' contributions

JK and DJR conceived of the study, carried out a detailed literature review, collected and presented the pertinent data. WOK and NOB participated in the study design and coordination and helped to draft the final manuscript. All authors read and approved the final manuscript.
